# Calpain inhibition in a transgenic model of calpastatin overexpression facilitates reversal of myocardial hypertrophy

**DOI:** 10.1002/ehf2.15250

**Published:** 2025-03-02

**Authors:** Gregor Sachse, Johanna Tennigkeit, Nikolaos Pagonas, Philipp Hillmeister, Ivo Buschmann, Martin Czolbe, Peter Nordbeck, Joachim Schmitt, Daniel Patschan, Oliver Ritter

**Affiliations:** ^1^ Department of Internal Medicine I University Hospital Brandenburg Brandenburg an der Havel Germany; ^2^ Brandenburg Medical School Neuruppin Germany; ^3^ Faculty of Health Sciences Joint Faculty of the Brandenburg University of Technology Cottbus, MHB Theodor Fontane, University of Potsdam Senftenberg Germany; ^4^ Department of Cardiology University Hospital Ruppin‐Brandenburg Neuruppin Germany; ^5^ Department of Internal Medicine I—Cardiology University Hospital Würzburg Würzburg Germany; ^6^ Comprehensive Heart Failure Center University Hospital Würzburg Würzburg Germany; ^7^ Department of Experimental Physics V Julius‐Maximilians‐University Würzburg Germany; ^8^ Research Center Magnetic Resonance Bavaria Würzburg Germany; ^9^ Institut für Pharmakologie und Toxikologie Universität Düsseldorf Düsseldorf Germany

**Keywords:** Heart failure, Myocardial hypertrophy, Signal transduction

## Abstract

**Aims:**

It was recently demonstrated that the intracellular signalling phosphatase calcineurin is subject to cleavage by the protease calpain, resulting in a truncated calcineurin fragment that is a strong inductor of myocardial hypertrophy. We now address the question of whether inhibition of calpain function in cardiomyocytes, and thereby prevention of calcineurin truncation, attenuates development of myocardial hypertrophy.

**Methods and results:**

We generated a transgenic mouse model with conditional cardiac calpastatin overexpression (CAST OE) and compared their cardiac hypertrophic response to angiotensin‐II (AngII) with that of non‐induced control animals. Angiotensin‐II osmotic mini‐pumps were removed 3 weeks after implantation and cardiac hypertrophy was re‐evaluated 3 weeks after pump removal. Induction of calpastatin overexpression resulted in 88% inhibition of calpain activity and suppressed calcineurin truncation. In CAST OE mice, basal phenotype and AngII‐induced myocardial hypertrophy were comparable with non‐induced controls (mean heart to body weight ratios ± SD in milligrams per gram: CAST OE, 4.8 ± 0.4; CAST OE + AngII, 7.1 ± 0.5; non‐induced, 4.9 ± 0.4; non‐induced + AngII, 7.2 ± 0.4). However, CAST OE mice demonstrated a complete reversal of hypertrophy when angiotensin‐II was removed, whereas hypertrophy persisted in non‐induced controls (CAST OE 5.0 ± 0.5; non‐induced 7.0 ± 0.4; *P* < 0.0001). Persistent hypertrophy in controls was accompanied by nuclear accumulation of truncated calcineurin and elevated activity of the Nuclear Factor of Activated T‐cells pathway. Moreover, we found that truncated calcineurin was insufficiently ubiquitinylated compared with its full‐length form and thus escaped degradation over several weeks in our in vivo experiments.

**Conclusions:**

Our data demonstrate that calpain‐mediated cleavage results in nuclear accumulation of a truncated, constitutively active and degradation‐resistant calcineurin isoform that sustains a long‐term myocardial hypertrophic response to angiotensin‐II beyond withdrawal of the stimulus. Cardiomyocyte specific calpain inhibition by transgenic calpastatin overexpression prevented the post‐stimulus myocardial hypertrophic response.

## Introduction

As post‐mitotic cells, cardiac myocytes require tight proteolytic control of regulatory protein levels to ensure cardiac homeostasis. Proteases and protein degradation systems like the ubiquitin–proteasomal system (UPS) or the autophagy‐lysosomal pathway remove defective regulatory proteins and control their turnover as a means of intracellular signalling.^1^, ^2^ Activity of the protease calpain is increased in a variety of cardiac pathological conditions, such as pressure overload,^3^ myocardial infarction^4^ or ischaemia reperfusion injury.^5^ Calpain activation has also been implicated in the pathogenesis of myocardial remodelling and heart failure.^6^

Previously, we found evidence that targeted proteolysis of calcineurin A (CnA) by calpain causes an increase in calcineurin activity in human myocardium.^7^, ^8^ This mode of activation complements reversible CnA activation by Ca^2+^, which initiates the CnA/Nuclear Factor of Activated T‐cells (NFAT) signalling cascade through the regulatory subunit calcineurin B and calmodulin.^9^ The CnA/NFAT cascade is one of the major pro‐hypertrophic signalling pathways in cardiac myocytes and requires sustained, elevated Ca^2+^ levels for activation. When activated by Ca^2+^/calmodulin, calcineurin directly binds and dephosphorylates NFAT family transcription factors, causing nuclear translocation of CnA/NFAT and initiation of hypertrophic gene expression. In contrast, calpain acts by proteolysis of the autoinhibitory domain (AID) of CnA, causing a constitutive increase in CnA activity even after removal of the hypertrophic stimulus.^8^ Calcineurin activation, both by Ca^2+^/calmodulin and by calpain‐mediated truncation, results in its translocation into the nucleus, which is required for full NFAT transcription factor activity.^10^, ^11^ Furthermore, we have demonstrated a compartmentalized signalling role for calcineurin as a nuclear sensor detecting local IP_3_ receptor‐mediated Ca^2+^ release from the nuclear envelope.^12^

Given its impact on the pivotal cardiac NFAT cascade, we hypothesized that calpain‐mediated proteolytic activation of calcineurin contributes substantially to developing and sustaining cardiac hypertrophic responses. To test this hypothesis, we generated a transgenic mouse line with conditional overexpression of calpastatin, a calpain inhibitor, and investigated the effect of calpain inhibition on the myocardial hypertrophic response to the stressor angiotensin‐II.

## Methods

### Generation of CAST OE mice

We used the Tet‐Off system (BD Biosciences, Heidelberg, Germany) for inducible expression of calpastatin in transgenic mice. A mouse strain expressing the regulatory protein tetracycline‐controlled transactivator (tTA) under control of the α‐MHC promoter [FVB.Cg‐Tg (Myh6‐tTA)6Smbf/J strain, The Jackson Laboratory, Bar Harbour, ME, USA] was crossed with a second strain [tetracycline‐responsive element (TRE)‐Cast] that we genetically engineered to express mouse calpastatin (Cast) under control of the TRE. To generate the TRE‐Cast strain, the murine Cast cDNA (kindly provided by M. Maki, Kyoto, Japan) was cloned into the pTRE‐6xHN vector (BD Bioscience, Franklin Lakes, NJ, USA). After microinjection of the pTRE‐6xHN‐Cast vector into fertilized FVB/N mouse oocyte pronuclei, offspring were screened for germline transmission by genotyping PCR. Genotyping primers were CATGTCCAGATCGAAATCGTC, CGCTGTGGGGCATTTTACTTT for the α‐MHC_tTA allele (450 bp fragment) and TTAACATGCTCCTGGGTGTG, CGGGGATCCTCTAGTCAGC for the TRE‐Cast allele (188 bp fragment). Experimental mice and their mothers were treated with 1 g/L doxycycline (Charles River, Sulzfeld, Germany) in drinking water, ad libitum, to suppress transgenic Cast expression during foetal and postnatal development. NFAT‐luciferase reporter transgenic mice were reported previously.^13^ All animal experimental procedures were reviewed and accepted by the local ethics committee and performed in accordance with the institutional guidelines for animal studies.

### Calpain assay

Frozen hearts were homogenized on ice in pre‐cooled, Ca^2+^‐free buffer (in mM: 25 imidazole‐HCl, 5 Cysteine, 1 EDTA; pH 7.5; all Sigma Aldrich, St. Louis, MO, USA). Lysates were then centrifuged (1 h, 4°C, 20 000 × *g*). The calpain‐specific proteolytic activity in the supernatant was assayed using the fluorogenic substrate Suc‐Leu‐Tyr‐AMC (‘CAS 94367‐20‐1 Calbiochem’, Merck, 208731). The samples were incubated with 5 mM CaCl_2_ and 50 μM Suc‐Leu‐Tyr‐AMC for 1 h at 30°C. As a non‐activated control, CaCl_2_ was replaced with 5 mM EDTA (pH 7.5). The reaction was stopped by the addition of alkali buffer (in mM: 250 glycine, 85 Na_2_CO_3_, 120 NaCl; pH 10.7; all Sigma Aldrich). Subsequently, the fluorescence F was measured in an LS 50 B spectrophotometer (Perkin Elmer) at 470 nm with excitation at 385 nm. Calpain‐specific proteolytic activity was calculated as follows: F_Ca2+_ − F_EDTA_.

### Magnetic resonance imaging and surgical models

Alzet miniosmotic pumps (no. 2002; Alza Corp, Mountain View, CA, USA) containing angiotensin‐II (2 mg/kg/day) were implanted subcutaneously into 2 month‐old mice after magnetic resonance imaging (MRI), then surgically removed after 3 weeks prior to an additional MRI. MRI was repeated a third time 3 weeks after pump removal. MRI, surgical procedures^14^ and anaesthesia protocols^15^ were conducted as described previously. Left ventricle (LV) remodelling was assessed in vivo by MRI using a 7‐Tesla Biospec MR Scanner (Bruker Biospin, Rheinstetten, Germany). Isoflurane inhalation was used for anaesthesia. In vivo MRI was performed using an ECG‐triggered fast low‐angle shot pulse sequence with the following imaging parameters: echo time, 1.5 ms; repetition time, 4.3 ms; field of view, 30 mm^2^; acquisition matrix, 128 × 128; slice thickness, 1.0 mm, and averages, 2–4. One stack of continuous short axis slices with 18–26 frames, dependent on heart rate, was acquired to cover the whole heart, spatially and temporally and determine diastolic and systolic three‐dimensional ventricular morphology and function. At midventricular level, an additional slice with a higher spatial resolution was acquired for exact determination of ventricular diameter, wall thickness and LV hypertrophy (acquisition matrix, 256 × 256; field of view, 30 mm^2^). Data analysis was performed using a custom semi‐automatic analysis routine as described before.^14^

### Luciferase assay

Calcineurin‐dependent NFAT activity was determined using whole heart samples from α‐MHC‐tTA × TRE‐CAST × NFAT‐Luc triple transgenic mice using a commercial luciferase assay system (Catalogue number E1500, Promega, Madison, WI). Cell lysis, sample preparation and luciferase assay were performed according to the manufacturer's protocol.

### Immunofluorescence and wheat‐germ agglutinin staining

Immunofluorescence staining on formalin‐fixed, paraffin‐embedded sections was performed as described before.^10^ The subcellular distribution of calcineurin was determined by immunostaining with anti‐CnA antibody (StressGen, SPA‐610). Myocyte cross‐sectional areas were measured in cardiac cryosections stained with wheat germ agglutinin–TRITC.

### Western blot and Co‐IP

Nuclear and cytoplasmic fractions were isolated using the NE‐PER kit (ThermoFisher, #78835). Sodium dodecyl sulfate–polyacrylamide gel electrophoresis (SDS‐PAGE), western blot and Co‐IP were performed as described previously.^14^ Tissues were lysed in precipitation assay buffer (1 × PBS, 1% IGEPAL CA‐630, Sigma, 0.5% sodium deoxycholate, Sigma/Complete™ EDTA‐free protease inhibitor mixture, Roche Diagnostics). After an initial preclearing step of 1 h at 4°C, antigens were bound to 5 μg of purified anti‐ubiquitin monoclonal antibody. Protein‐antibody complexes were precipitated using a mixture of 50 μL of protein A and 50 μL of protein G‐Sepharose beads for 1 h at 4°C. After four washing steps with precipitation assay buffer and one with 50 mM Tris, pH 8.0, proteins were eluted with 100 μL of sample buffer, then separated using SDS‐PAGE and blotted to PVDF membranes for chemiluminescence detection (ECL system, Amersham Pharmacia Biotech). Antibodies: anti‐6xHN (Clontech, #8940‐1); anti‐GAPDH (Abcam, Ab125247); anti‐Histone H3 (St. Cruz, sc‐517576); anti‐ubiquitin (Ubi‐1, ThermoFisher, #13‐1600); anti‐CnA (StressGen, SPA‐610).

### Preparation of neonatal rat cardiomyocytes and expression constructs

Derivatives of calcineurin A (isoform CnAβ), containing N‐terminal enhanced green fluorescent protein (EGFP), were generated using the mammalian expression vector pEGFP‐C3 (BD Biosciences/Clontech). The following C‐terminal truncated mutants were amplified by PCR and cloned into the XbaI and XhoI sites of the pEGFP‐C3 plasmid: CnA(1‐525), CnA(1‐485), CnA(1‐465), CnA(1‐465,K456R), CnA(1‐445) and CnA(1‐425). Adenoviral vector AdCnA isoforms were generated by subcloning cDNAs into the pShuttle‐CMV vector, then incorporated into virus particles using the AdEasy XL Adenoviral Vector system (Stratagene). Neonatal rat cardiomyocytes of Wistar rats (Harlan‐Winkelmann, Borchen, Germany) were isolated as described previously,^10^ then infected with adenoviruses at a multiplicity of infection of 25–50.

### Statistical analysis

Unless stated otherwise, results are presented as scatter plots with means ± SEM. Statistical analysis was performed using GraphPad PRISM software. ANOVA and unpaired Welch's *t*‐test were used unless noted otherwise. As post‐hoc correction for multiple comparisons, the significance criterion of *P* < 0.05 was adjusted with the Bonferroni method.

## Results

### Transgenic, conditional overexpression of calpastatin inhibited calpain activity in primary heart tissue

**Figure 1:**
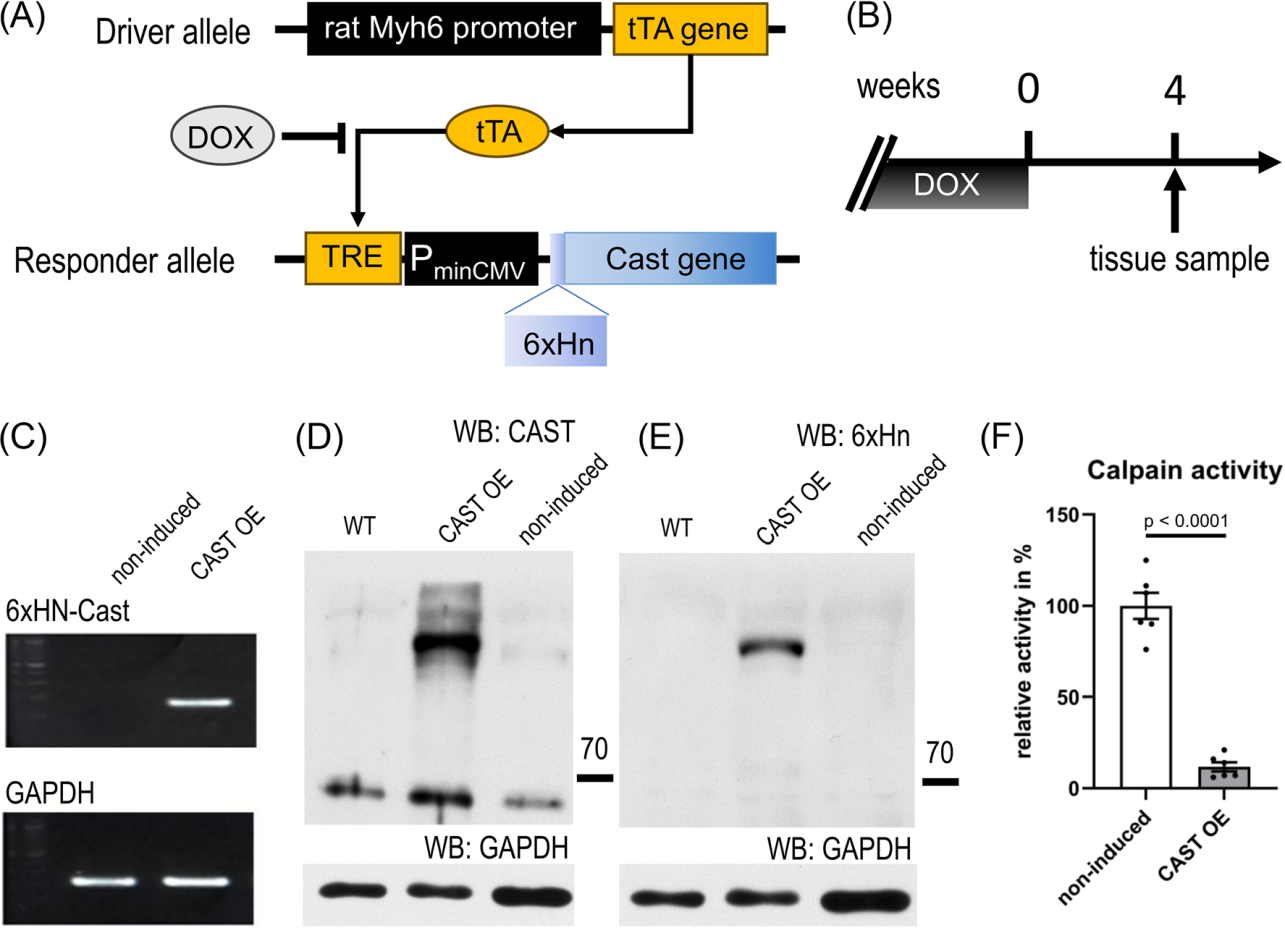
Inducible cardiac‐specific expression of calpastatin and inhibition of calpain enzymatic activity in α‐MHC‐tTA × TRE‐Cast double‐transgenic mice (CAST OE mice). (A) Schematic representation of the transgenic constructs used to enable inducible calpastatin expression in the heart. Cast, calpastatin mini‐gene; DOX, doxycycline; P_minCMV_, minimal CMV promoter; TRE, tetracycline‐responsive element; tTA, tetracycline‐controlled transactivator; 6xHn, N‐terminal 6x‐His tag. (B) Timeline for induction of cardiac calpastatin expression. Samples were tissue lysates from hearts. Representative reverse transcription polymerase chain reaction results (C) and representative western blots (WB) for total (D) and 6xHN‐tagged (E) calpastatin, showing induction of transgenic calpastatin expression in heart. Loading control was GAPDH. WT, wild type. (F) Sample calpain activity, determined using a synthetic substrate (Suc‐Leu‐Tyr‐AMC) for a colour reaction with detection at 470 nm. Data are shown as individual data points, means and SEM.

In earlier studies, we showed that calcineurin is activated through targeted proteolysis by calpain. Truncated calcineurin is constitutively active and strictly localizes to the cell's nucleus.^7^ This is because truncation causes loss of the AID of calcineurin, loss of the nuclear export sequence and exposes a previously hidden nuclear localization signal motif.^12^ However, the role and significance of truncated calcineurin in the context of myocardial hypertrophy remains to be determined. Therefore, we generated transgenic mice with cardiac‐specific expression of calpastatin (an inhibitor of calpain) under control of the α‐MHC promoter (*Figure* 1A). The tetracycline‐responsive‐element (TRE) conditional system was chosen because constitutive loss of myocardial calpain activity by forced overexpression of calpastatin had resulted in progressive dilated cardiomyopathy and degeneration of sarcomeres in an earlier mouse model.^16^ In the TRE conditional system, absence of doxycycline permits α‐MHC promoter‐driven tetracycline activator protein (tTA) to switch on a TRE‐calpastatin minigene (TRE‐Cast) in cardiomyocytes of α‐MHC‐tTA × TRE‐Cast double‐transgenic mice (*Figure* 1A). Constitutive doxycycline treatment resulted in complete suppression of ectopic calpastatin in the heart of double‐transgenic mice, which was reversed after omitting doxycycline for 4 weeks (*Figure* 1B), as shown by reverse transcriptase polymerase chain reaction (*Figure* 1C) and immunoblot detection of calpastatin (*Figure* 1D) and of the ectopic calpastatin 6xHN tag (*Figure* 1E). Several mature forms of calpastatin exist, of which the most prominent are the full‐length form, with a calculated MW of 68 kDa (but runs at an apparent MW of 105–110 K on SDS–PAGE), and the second‐longest form (46 kDa, apparent MW of 68 kDa).^16^ While there was some expression of 46 kDa calpastatin in hearts of wild‐type and non‐induced double‐transgenic mice, only induced CAST OE mice showed full‐length calpastatin expression. Moreover, calpastatin overexpression at 4 weeks without doxycycline caused an 88% inhibition of myocardial calpain activity (100% ± 7% vs. 12% ± 2%) (*Figure* 1F).

### Inhibition of calpain affected the regression of myocardial hypertrophy, with no effect on its development

**Figure 2:**
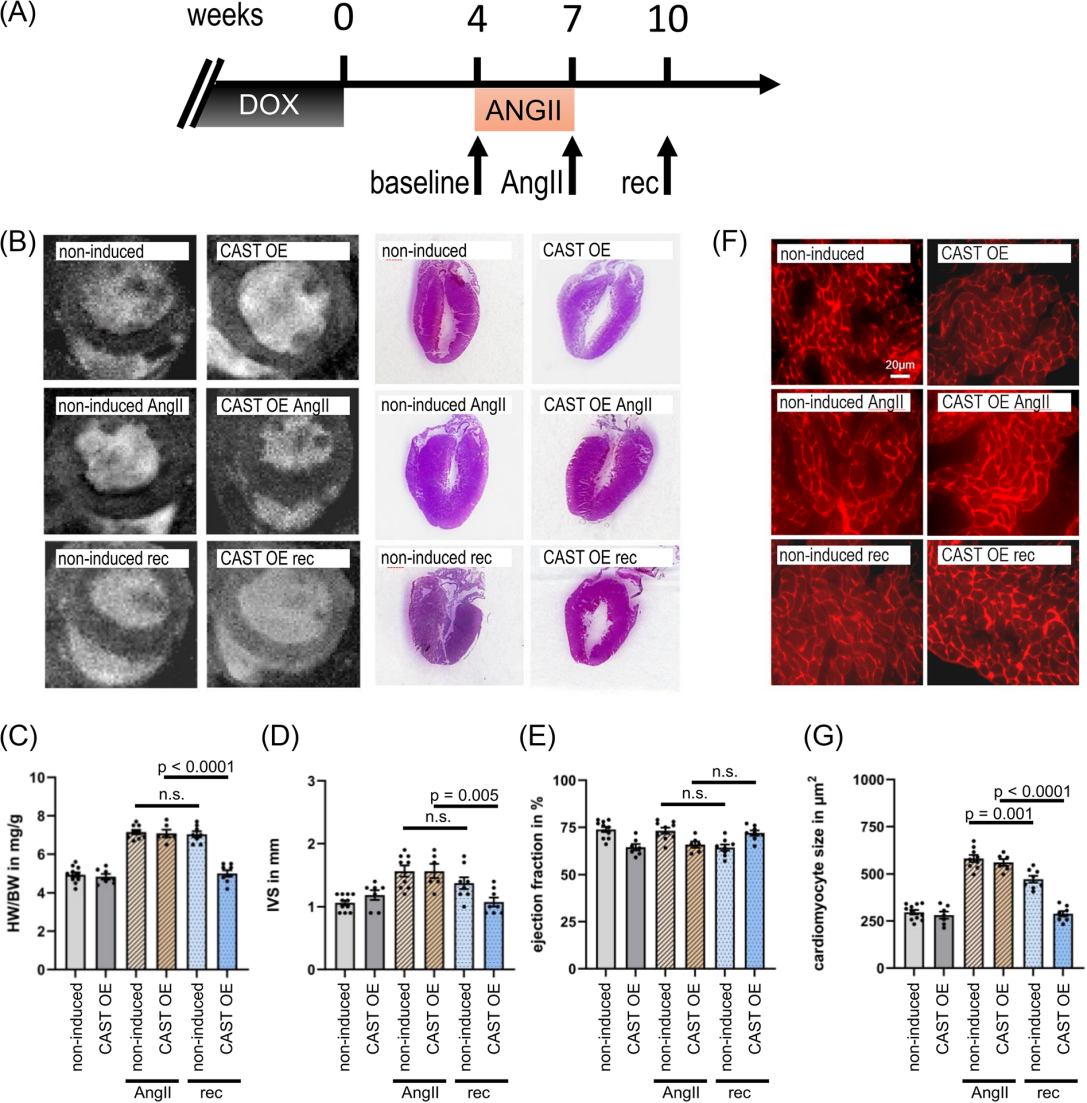
Angiotensin‐II‐driven cardiac hypertrophy and recovery period in cardiac calpastatin overexpressing mice and DOX controls. (A) Induction of calpastatin expression at time point 0 by removal of doxycycline (DOX) in the calpastatin overexpressing group (CAST OE). Non‐induced littermates remained on doxycycline. CAST OE and non‐induced were challenged with AngII osmotic mini‐pumps for 3 weeks (+AngII), then recovered for three more weeks after pump removal (rec). MRI imaging on live animals was performed at Weeks 4, 7 and 10 as indicated. Examinations were performed in a time frame of 2 days. (B) Representative cardiac short axis MRI images and representative HE‐stained longitudinal heart sections to assess LV hypertrophy, for all groups. (C) Heart–body weight ratio (HW/BW). (D) Diameter of the interventricular septum (IVS). (E) Ejection fraction. IVS and EF were determined using 7 T high‐field MRI. (F) Cardiac cryosections, membrane‐stained with wheat germ agglutinin‐TRITC. (G) Average cardiac myocyte cross‐sectional area (>100 cells per heart). Data are shown as individual data points, means and SEM.

Three weeks of AngII stimulation using osmotic mini‐pumps resulted in the induction of myocardial hypertrophy both in doxycycline controls and in mice with calpastatin overexpression/calpain inhibition (*Figure* 2A,B). While there was no difference in body mass, heart–body weight ratios increased significantly (*Figure* 2B,C), as well as the diameter of the interventricular septum IVS, as assessed by MRI (*Figure* 2B,D). Ejection fractions stayed the same (*Figure* 2E). The increase in heart mass was accompanied by increased cardiomyocyte cross‐sectional area (*Figure* 2F,G).

While CAST OE and non‐induced hearts responded similarly to AngII treatment, there were significant differences after withdrawal of AngII stimulation. 3 weeks after removal of AngII mini‐pumps, non‐induced animals with intact calpain function showed substantial hypertrophy (*Figure* 2B–D), in agreement with our previous report on the AngII cardiac hypertrophy mouse model.^12^ However, we observed a complete reversal of myocardial hypertrophy in animals with calpastatin overexpression/calpain inhibition (*Figure* 2B–D). The cross‐sectional area of cardiomyocytes decreased substantially after the 3 week recovery period in induced CAST OE mice (from 560 ± 49 μm^2^ to 289 ± 39 μm^2^; *P* < 0.0001), in contrast to a distinctly more limited reduction in non‐induced mice (from 581 ± 37 μm^2^ to 473 ± 33 μm^2^; *P* < 0.001) (*Figure* 2G).

### Inhibition of calpain affects calcineurin nuclear export and NFAT transcriptional activity

**Figure 3:**
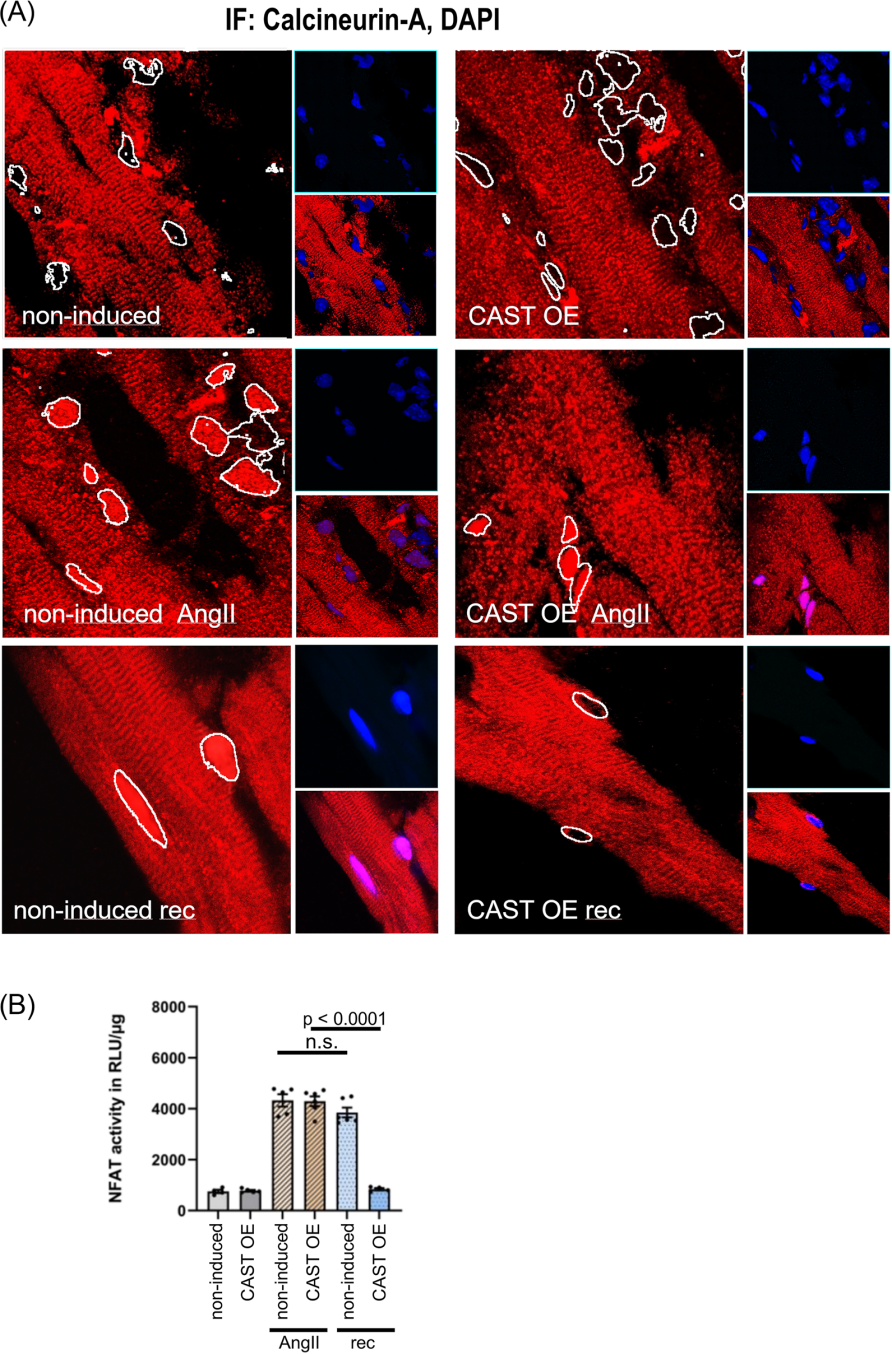
Calcineurin subcellular localization and NFAT activity in CAST OE cardiac myocytes and non‐induced controls. (A) Heart sections from mouse experimental groups as shown in *Figure* 2, immunofluorescence‐stained with anti‐CnA antibodies (red) and DAPI nuclear stain (blue). Nucleus outlines were highlighted (white) by edge detection filter based on the DAPI channel. (B) Calpastatin transgenic animal were crossbred with NFAT‐Luc mice to detect calcineurin NFAT activity. NFAT activity was determined by luciferase assay in heart lysates from calpastatin transgenic animals that were crossbred with NFAT‐Luc mice at the same time points as shown in *Figure* 2A. Data are shown as individual data points, means and SEM.

Next, we performed immunofluorescence staining of heart sections to determine whether the differences in hypertrophic response between CAST OE mice and non‐induced controls were associated with altered nuclear translocation of calcineurin (*Figure* 3). Calpastatin overexpression did not affect CnA location at baseline. Localization was predominantly sarcomeric due to MLP‐dependent anchorage to the Z‐disc,^17^ with no CnA detected in the nucleus. In both CAST OE mice and non‐induced controls, we found nuclear accumulation of calcineurin A 3 weeks after AngII stimulation. In CAST OE animals with calpain inhibition, calcineurin nuclear accumulation was reversed after removal of AngII and returned to baseline, whereas in non‐induced mice with intact calpain function, there was a sustained nuclear accumulation of calcineurin even after removal of AngII (*Figure* 3A).

To investigate whether nuclear calcineurin localization correlated with NFAT transcriptional activity, calpastatin transgenic animals were crossbred with NFAT‐Luc mice. Calpastatin overexpression at baseline did not affect NFAT luciferase activity [757 ± 61 RLU/μg^−1^ in non‐induced mice vs. 782 ± 71 RLU/μg^−1^ in CAST OE mice (n.s.)]. AngII stimulation increased NFAT Luciferase activity to a similar extent in both non‐induced and CAST OE mice [4328 ± 362 RLU/μg^−1^ vs. 4294 ± 402 RLU/μg^−1^ (n.s.)]. In CAST OE animals with calpain inhibition, NFAT Luciferase activity returned to baseline after removal of AngII (4294 ± 402 RLU/μg^−1^ vs. 846 ± 40 RLU/μg^−1^, *P* < 0.0001), whereas intact calpain function in non‐induced controls resulted in sustained NFAT luciferase activity also after removal of AngII (4328 ± 362 RLU/μg^−1^ vs. 3847 ± 440 RLU/μg^−1^, n.s.) (*Figure* 3B).

### Truncated calcineurin escaped UPS‐mediated degradation

We demonstrated previously that both constitutively active truncated calcineurin and full length calcineurin are translocated into the nucleus upon angiotensin‐II challenge.^7^ Our observation that removal of nuclear calcineurin following AngII withdrawal is faster in calpastatin overexpressing animals (*Figure* 3A,B) suggests that nuclear export or degradation of truncated calcineurin could be affected.

**Figure 4:**
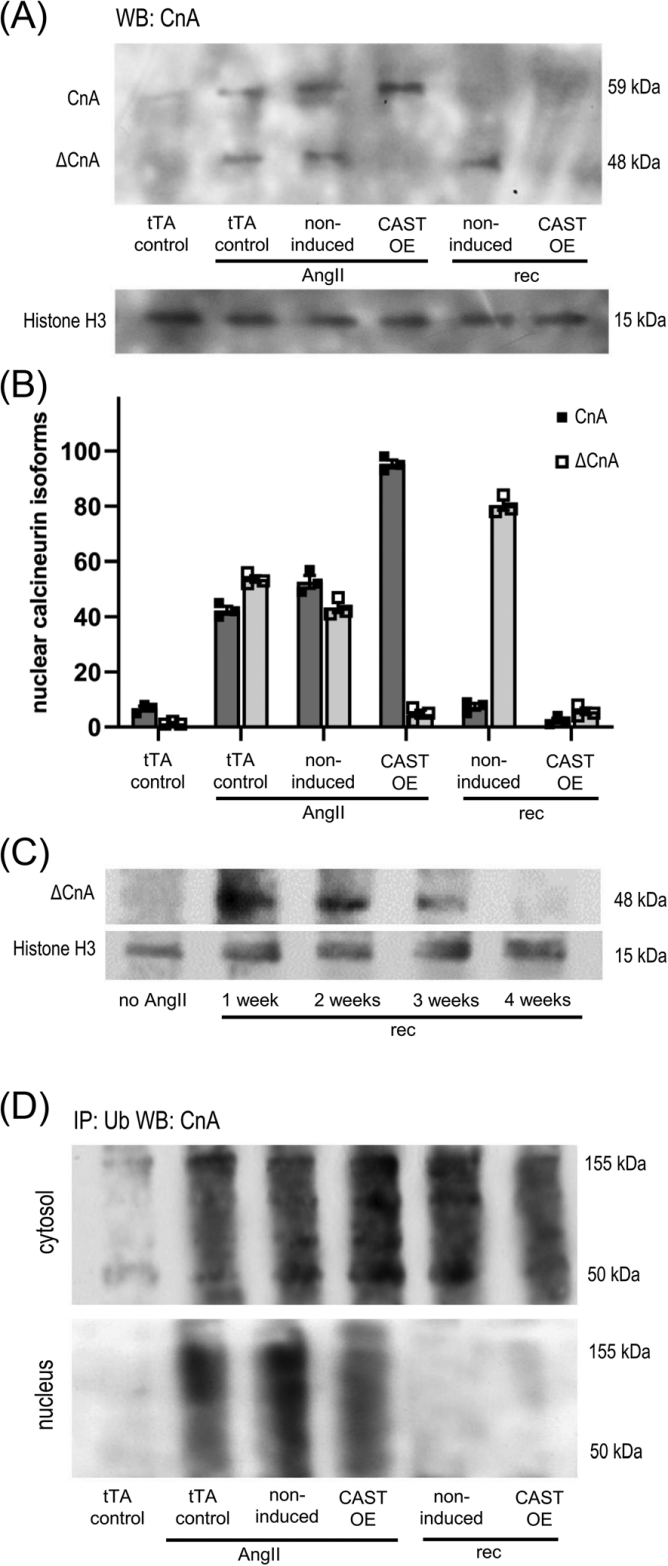
Subcellular localization and degradation of truncated versus full‐length calcineurin A. (A) Western blot (WB) detecting full‐length (CnA, 59 kDa) and truncated (ΔCnA, 48 kDa) calcineurin (CnA) in the nuclear heart tissue fractions of tTA control, calpastatin overexpressing (CAST OE) and non‐induced mice that were challenged with angiotensin‐II (AngII) or after 3 weeks into recovery phase (rec), as shown in *Figure* 2A. (B) Relative densitometric quantification of the calcineurin isoforms detected by immunoblot, as shown in Part (A). (C) Time course of nuclear ΔCnA content in calpastatin‐induced mice after removal of AngII. For each time point, homogenates of three hearts were pooled. (D) Cytosolic and nuclear fractions of pooled heart homogenates, immunopurified with anti‐ubiquitin antibody‐coupled beads (IP: Ub), followed by anti‐CnA western blot.

We, therefore, measured cardiac expression levels of full length (CnA, 59 kDa) and truncated (ΔCnA, 48 kDa) calcineurin A in calpastatin overexpressing mice and non‐induced controls at two time points: 3 weeks after implantation of AngII mini‐pumps (AngII) and 3 weeks after the subsequent removal of the pumps (rec) (*Figure* 4A). Quantitative analysis showed low baseline CnA expression, and strong induction of CnA/ΔCnA expression by AngII (*Figure* 4B). Calpastatin suppression of calpain activity in CAST OE animals prevented CnA truncation, with total levels of nuclear CnA comparable with controls. After removal of AngII, full length calcineurin was absent from the nucleus (*Figure* 4B) while ΔCnA remained detectable for up to 4 weeks (*Figure* 4B,C).

We also performed anti‐ubiquitin immunopurification followed by anti‐CnA western blot detection to assess proteasome‐mediated CnA removal in heart lysate cytosolic and nuclear fractions (*Figure* 4D). In calpastatin‐overexpressing mice as well as in non‐induced controls, ubiquitinylated CnA/ΔCnA was present both in cytosol and nuclei under AngII stimulation. In contrast, ubiquitinylated CnA/ΔCnA was absent from nuclei after removal of AngII and only detectable in the cytosol. Because cardiac nuclei of non‐induced mice contain substantial amounts of ΔCnA after removal of AngII (*Figure* 4B, non‐induced rec), this suggests that ΔCnA within the nucleus escapes ubiquitinylation.

### Calcineurin truncation prevents ubiquitinylation

**Figure 5:**
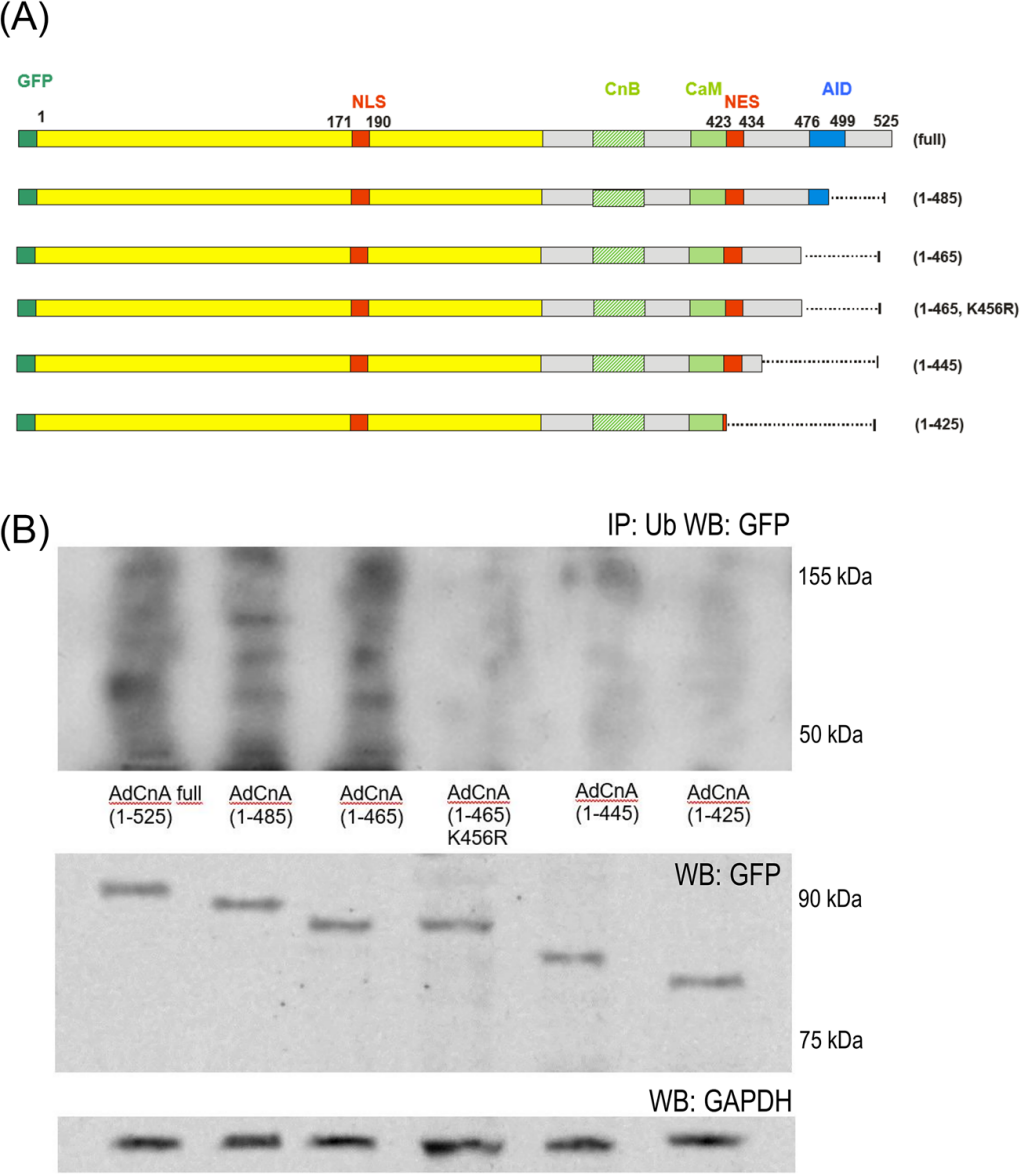
Mapping of calcineurin A ubiquitinylation sites. (A) Schematic representation of calcineurin adenoviral vectors with progressive C‐terminal truncation. All constructs were N‐terminally fused to a GFP tag (not to scale). K456R indicates substitution of Lys with Arg at residue 456. AID, autoinhibitory domain; CaM, calmodulin binding domainCnB, calcineurin B binding domain; NES, nuclear export signal; NLS, nuclear localization sequence. (B) Lysates of neonatal rat cardiomyocytes transfected with the vectors shown in Part (A), immunopurified with anti‐ubiquitin antibody‐coupled beads (IP: Ub), followed by anti‐GFP western blot. ‘WB: GFP’ indicates expression control of calcineurin isoforms. ‘WB: GAPDH’ indicates the loading control.

We transfected neonatal rat cardiomyocytes with different length CnA isoforms to find C‐terminal CnA domains crucial for ubiquitinylation (*Figure* 5A). CnA isoforms longer than 1‐465 could be ubiquitinylated. Isoforms truncated at residue 424 (as calpain does) escaped ubiquitinylation. The calcineurin isoform 1‐465 could be rescued from ubiquitinylation by substitution of Lys‐456 with Arg (*Figure* 5B).

## Discussion

The data presented here provide deeper insight into the role of proteolytic systems in myocardial remodelling, specifically how control of calpain proteolytic activity by the specific endogenous inhibitor calpastatin affects the reversal of myocardial hypertrophy after removal of a pro‐hypertrophic stimulus. Results from our conditional CAST OE mouse model differ significantly from previous models based on constitutive calpastatin overexpression. In one model, constitutive cardiac calpastatin overexpression resulted in a slowly progressive dilated cardiomyopathy associated with accumulation of intracellular protein aggregates.^16^ In another model, mice with systemic constitutive overexpression of calpastatin were phenotypically normal at baseline but showed a blunting of NF‐κB signals under AngII stimulation and thus had reduced cardiac and vascular hypertrophy, as well as perivascular fibrosis.^18^ Antihypertrophic effects of the systemic overexpression model were broader compared with our CAST OE model, possibly due to constitutive expression from birth and the effect of calpastatin overexpression on endothelial function^18^, ^19^ and the recruitment of cardiac mononuclear immune cells.^20^

**Figure 6:**
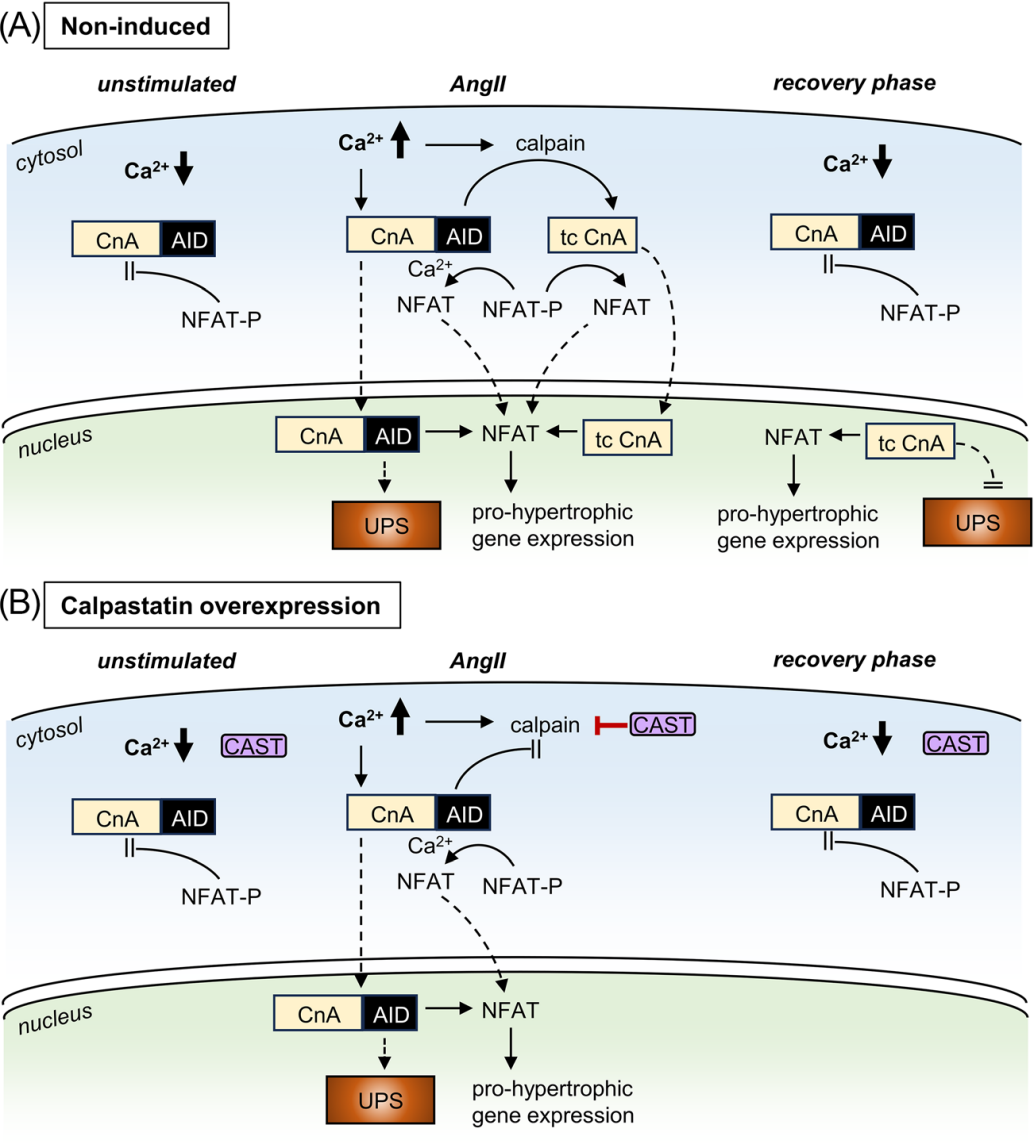
Calpastatin‐dependent changes in calcineurin signalling during and after AngII stimulation. (A) Cardiomyocyte calcineurin‐NFAT signalling before (‘unstimulated’), during angiotensin‐II treatment (‘AngII’) and after angiotensin‐II removal (‘recovery phase’); in non‐induced double‐transgenic mice. (B) Same as in Part (A), but for induced CAST OE mice. Here, calpain enzymatic activity in cardiomyocytes is supressed by conditional overexpression of calpastatin (CAST). AID, autoinhibitory domain; CnA, calcineurin A; NFAT, nuclear factor of activated T cells; NFAT‐P: phosphorylated NFAT; tc CnA, truncated CnA; UPS, ubiquitin–proteasome system. Dashed arrows denote translocation.

In our CAST OE mice, we found that long‐term angiotensin‐II stimulation caused a pronounced cardiac hypertrophic response that persisted weeks after the end of the stimulus (*Figure* 2), confirming our previous report using the AngII cardiac hypertrophy mouse model.^12^ We now present novel data, demonstrating that the persistent hypertrophic response after the stimulus is carried by truncated calcineurin A inside the cardiomyocyte nucleus. In control animals (*Figure* 6A), increased workload and sustained Ca^2+^ release promoted calcineurin truncation by the protease calpain, thereby removing both the AID of calcineurin and the nuclear export signal (NES) (protein domains are shown in *Figure* 5A). This rendered the remaining calcineurin constitutively active and nuclear, as demonstrated previously.^7^, ^12^ In calpastatin overexpression mice, however (*Figure* 6B), calpastatin inhibition of calpain enzymatic activity prevented calcineurin A truncation, which did not affect the acute cardiac hypertrophic response but greatly facilitated recovery after the end of angiotensin‐II stimulation (*Figure*s 2 and 3). This suggests that UPS‐mediated degradation is crucial for the regulation of calcineurin‐mediated NFAT signalling, in line with earlier evidence that UPS inhibition can upregulate calcineurin NFAT signalling, causing myocardial hypertrophy.^21^, ^22^

Moreover, we found that persistent presence of truncated calcineurin A in the cardiomyocyte nucleus after AngII stimulation is linked not only to the loss of the calcineurin nuclear export signal but also renders calcineurin resistant to degradation by the ubiquitin–proteasome system (*Figure*s 4 and 5). This is essential for the persistent NFAT activity that we observed in post‐AngII non‐induced animals (*Figure* 3B) because in the absence of nuclear calcineurin activity, NFAT is rapidly re‐phosphorylated and leaves the nucleus within minutes.^23^ It was previously demonstrated that calcineurin can be degraded by UPS after ubiquitinylation via the E3 ligase atrogin.^24^ The atrogin binding site on calcineurin is not truncated by calpain. However, in our neonatal rat cardiomyocyte experiments, we found that calcineurin A ubiquitinylation depended exclusively on C‐terminal lysine residues that are absent in truncated calcineurin, likely explaining its resistance to UPS degradation (*Figure* 5). N‐terminal lysines in the calcineurin B binding domain (position 369) and in the calmodulin binding domain (403, 409, 415) appear not to be substrates for ubiquitinylation.

Our data show that CnA is slowly removed from the cell nucleus even in the absence of ubiquitinylation (*Figure* 4C). In light of the detrimental effect of nuclear CnA, targeting the removal of nuclear CnA, as well as the CnA‐NFAT interaction specifically in the nuclear compartment, could be powerful therapeutic strategies. However, CnA removal might be a passive process, as its size does not exclude diffusion through the nuclear pore.^25^ This would then make it accessible to degradation by the autophagy–lysosome pathway (ALP). Interestingly in this context, CnA signalling itself is involved in ALP activation, as detected following cardiac proteasome malfunction.^26^

Our findings highlight that calcineurin activation by calpain cleavage causes (patho)physiological effects that Ca^2+^‐dependent activation does not. These results suggest a mechanism whereby increased myocardial workload and persistent Ca^2+^ stimulation cause a rise in the intranuclear calcineurin pool that consists of, in equal parts, full‐length calcineurin that can shuttle between nucleus and cytosol and truncated calcineurin that is nucleus bound. The truncated and nuclear CnA fraction then sustains the hypertrophic response beyond removal of the hypertrophic stimulus. This may be an effective way by which cardiomyocytes can integrate neurohumoral signals via Ca^2+^‐dependent mechanisms without being distracted by cytosolic beat‐to‐beat Ca^2+^ transients. Moreover, this may act synergistically with the high Ca^2+^ affinity of calcineurin, which is ~100‐fold higher than that of CaMKII.^12^ Besides these nuclear Ca^2+^ sensing properties, calcineurin is also important in cardiac mechanotransduction via Z‐disc proteins (Lmcd1/Dyxin, MLP, calsacrin).^27^ Combining these observations, we propose calcineurin as a compartmentalized Ca^2+^ sensor that is optimized to respond to modest or short acting elevations of Ca^2+^ and integrate them into a sustained NFAT‐signalling response. In a clinical context, this would be relevant to pathologies like paroxysmal atrial fibrillation, that are characterized by short term intervals of atrial (tachy‐) arrhythmias, accompanied by alterations in Ca^2+^ homeostasis.^28^ An involvement in physiological cardiac hypertrophy in response to hour‐long episodes of exercise is also plausible. Moreover, this mechanism could help explain how treatments that successfully prevent onset of hypertrophy might still fail to reverse it once established.

## Conflict of interest statement

None declared.

## Funding

This work was supported by the Federal Ministry of Education and Research (Germany) (IFB ‘heart failure’, projects B2, C2; grant VIP0014) and the German Research Foundation (grant Ri1085/4‐1) to O. R.
